# Society Meetings

**Published:** 1910-11

**Authors:** 


### SOCIETY MEETINGS

THE New York and New England Association Railway Surgeons will hold its twentieth annual session at the Hotel Astor, Broadway and Forty-Fourth Street, New York City, November 3-4,

1910, under the presidency of Dr. L. M. Bingham, of Burlington, Vt. An excellent program has been arranged. Dr. John B. Deaver, of Philadelphia, will deliver the "Address on Surgery" November 3d, at 12 o'clock. A cordial invitation is extended to railway officials, railway surgeons, and to the medical profession to attend this session of the association. Dr. George Chaffee, 338 47th Street, Brooklyn, N. Y., is the corresponding secretary.

---

THE Buffalo Academy of Medicine held meetings during the month of October, 1910, as follows:

*Section on Surgery.*—Tuesday evening, October 4. Program: Inflammation of the verumontanum, John A. Hawkins, Pittsburg; Personal experiences with genitourinary cases, including genital tuberculosis and inflammatory prostatic conditions, Frederick W. Robbins, Detroit; discussion was opened by James A. Gardner.

*Section on Medicine.*—Tuesday evening, October 11. Program: Purins in diathesis, in disease, in treatment, Henry R. Hopkins; Cardiac nutrition, Eli H. Long; Chylous cyst of the mesentery, A. L. Benedict.

*Section on Pathology.*—Tuesday evening, October 18. Program: Pneumonia treated with pneumococcic vaccine, R. C. Rosenow, Chicago.

*Section on Obstetrics and Gynecology.*—Tuesday evening, October 25. Program: The choice of time for operation in pelvic inflammation of tubal origin, F. F. Simpson, Pittsburg.

